# Application of three-dimensional (3D) reconstruction in the treatment of video-assisted thoracoscopic complex segmentectomy of the lower lung lobe: A retrospective study

**DOI:** 10.3389/fsurg.2022.968199

**Published:** 2022-09-29

**Authors:** Xinyu Wang, Qing Wang, Xindi Zhang, Hang Yin, Yujie Fu, Min Cao, Xiaojing Zhao

**Affiliations:** Department of Thoracic Surgery, Renji Hospital, Shanghai Jiao Tong University School of Medicine, Shanghai, China

**Keywords:** 3D reconstruction, complex segmentectomy, ground-glass nodules (GGN), Japan Clinical Oncology Group (JCOG), lung cancer

## Abstract

**Background:**

An increasing number of lung ground-glass nodules (GGNs) have been detected ever since low-dose computer tomography started growing in popularity. Three-dimensional (3D) reconstruction technology plays a critical role in lung resection, especially in segmentectomy. In this study, we explore the role of 3D reconstruction in thoracoscopic complex segmentectomy of lower lung lobe.

**Methods:**

A total of 97 patients who underwent complex segmentectomy of lower lung lobe from January 2021 to March 2022 were retrospectively analyzed. We divided these patients into a 3D group (*n* = 42) and a routine group (*n* = 55) based on preoperative 3D reconstruction or without this procedure. The demographics of patients and GGNs were collected and perioperative outcomes were compared between the two groups.

**Results:**

All of the baseline characteristics between the groups were comparable (all *P* > 0.05). There was no 30-day postoperative mortality and conversion in the two groups. The operation time of the 3D group was significantly shorter than that of the routine group (111.4 ± 20.8 min vs. 127.1 ± 32.3 min, *P* = 0.007). The number of stapler reloads during surgery in the 3D group was less than that in the routine group (9.0 ± 2.2 vs. 10.4 ± 2.6, *P* = 0.009). The rate of air leakage on postoperative days 1–3 was lower in the 3D group (11.9% vs. 30.9%, *P* = 0.027). In addition, the resection margins of all patients in the 3D group were adequate, while four patients in the routine group had inadequate resection margins, although there was no statistically significant difference (*P* = 0.131). Intraoperative blood loss, postoperative drainage, postoperative hospital stay, pneumonia/atelectasis, and hemoptysis were similar between the two groups.

**Conclusions:**

For performing complex segmentectomy of the lower lung lobe, the procedure of 3D reconstruction may shorten the operation time, decrease the number of stapler reloads, prevent postoperative air leakage, and guarantee a safe surgical margin. Therefore, 3D reconstruction is recommended for complex segmentectomy of the lower lung lobe.

## Introduction

With the development and growing popularity of low-dose computer tomography (LDCT) screening, an increasing number of lung ground-glass nodules (GGNs), including pure GGNs (pGGNs) (GGNs without solid components) and mixed GGNs (mGGNs) (GGNs with solid components), have been detected (1). Although the majority of GGNs are benign lesions, some GGNs are pathologically confirmed to be malignancies, requiring surgical resection (2). The Japan Clinical Oncology Group 0804 (JCOG0804) has proved that sublobar resection (wedge resection or segmentectomy) is safe and feasible for resecting noninvasive lung cancers featured with a diameter ≤2.0 cm and a consolidation-to-tumor ratio (CTR) ≤0.25 (3). More recently, JCOG0802 has shown the benefits of segmentectomy vs. lobectomy in terms of the overall survival rate of patients with clinical stage IA lung cancer with a tumor diameter ≤2 cm and CTR >0.5, suggesting that segmentectomy may be the standard surgical procedure for these patients (4). Therefore, the indication of segmentectomy for lung cancer is extending.

Segmentectomy could be divided into simple segmentectomy and complex segmentectomy according to the surgical procedures and intersegmental planes. Simple segmentectomy includes right S6, left S6, left S1 + 2 + 3, and left S4 + 5, which is completed by creating only one linear intersegmental plane. Complex segmentectomy, such as the resection of the right S2, left S3, and bilateral S9, has excluded the above simple segmentectomy by creating multiple intersegmental planes (5). For simple segmentectomy, routine preoperative high-resolution CT (HRCT) images are adequate for surgery. On the contrary, complex segmentectomy requires more preoperative plans and intraoperative skills, beyond the information that routine CT could provide (6). It is urgent to apply some assistant technologies, such as preoperative three-dimensional (3D) simulation technology.

Since the emergence of 3D technology, it is widely acknowledged that 3D reconstruction is playing a critical role in lung resection, especially in segmentectomy. During the performance of segmentectomy, it is challenging and time-consuming for identifying the target segment structures accurately. A 3D lung reconstruction includes the simulation of pulmonary vessels and the space between the lesion and the adjacent tissues, which provides substantial support for locating the target segment structures.

Although some studies have proved the effectiveness of 3D reconstruction in segmentectomy, most of them include all types of segmentectomy (e.g., simple and complex segmentectomy, upper and lower lung lobe segmentectomy) in their studies (7–14). As a result, it is tough to control multiple confounding factors under such circumstances.

The anatomic variation of the lower pulmonary structure is more prevalent than that of the upper lung (15). We assume that 3D reconstruction could provide greater assistance in the field of complex segmentectomy of the lower lung lobe. This study is aimed to explore the role of 3D reconstruction in thoracoscopic complex segmentectomy of the lower lung lobe.

## Materials and methods

### Patient selection

**Figure 1:**
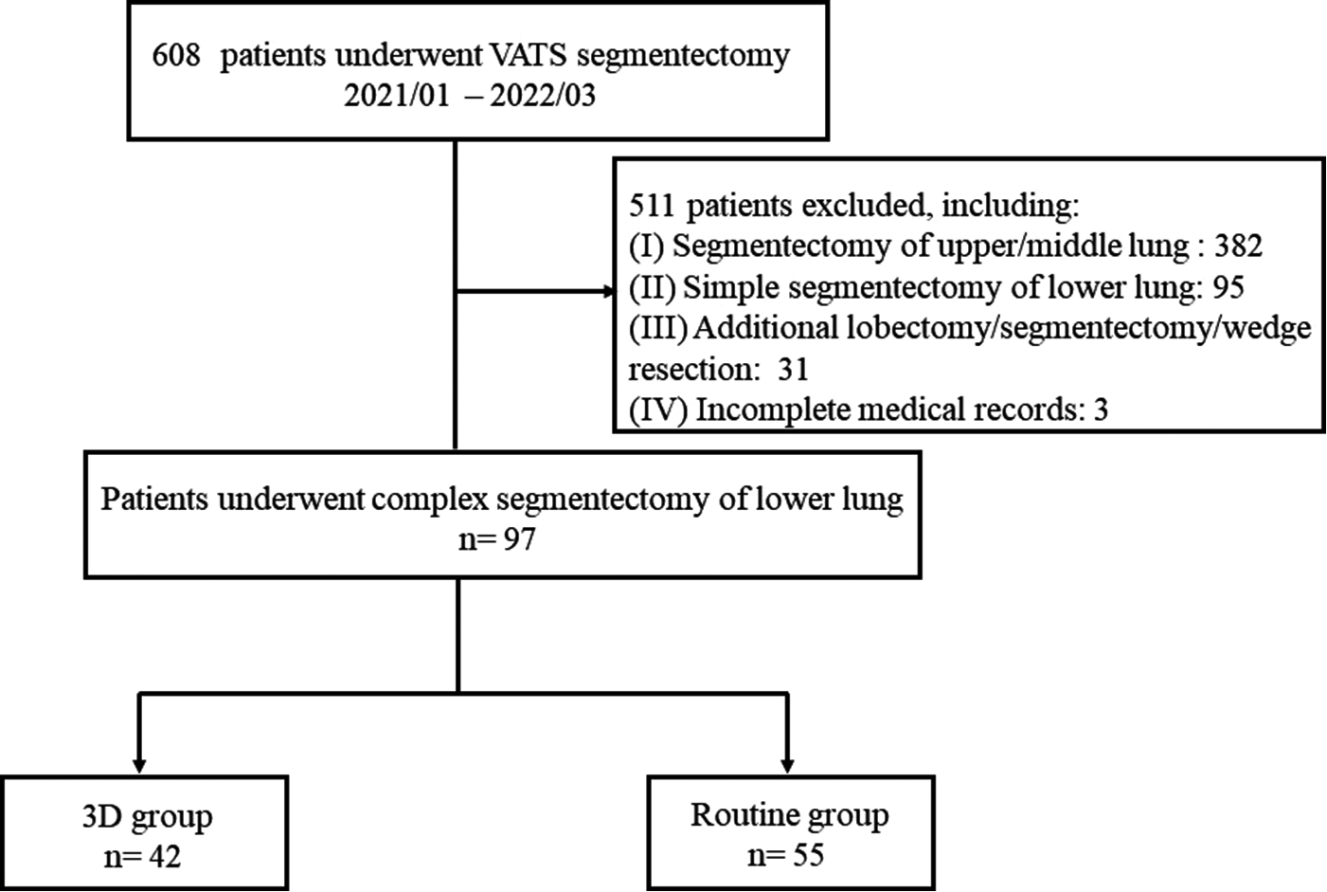
Selection algorithm of patients in this study.

Figure 1 demonstrates the selection algorithm of our patients in this study. A total of 608 consecutive patients who underwent video-assisted thoracic surgery (VATS) segmentectomy from January 2021 to March 2022 were retrospectively analyzed. VATS segmentectomy was performed in our center according to the following criteria: (I) peripheral pGGNs or mGGNs, highly suspicious for malignancy and (II) ≤2 cm in diameter. Complex segmentectomy was further screened. Complex segmentectomy of the lower lung lobe was defined as all segmentectomies other than S6 resection, including S7 (right), S8, S9, S10, and a combined resection of segmentectomy (S8 + 9, S9 + 10, etc.). Patients were excluded if they had the following: (I) segmentectomy of the upper or middle lung (*n* = 382); (II) simple segmentectomy of the lower lung lobe (*n* = 95); (III) additional lobectomy/segmentectomy/wedge resection (*n* = 31); (IV) incomplete medical records (*n* = 3). Eventually, 97 patients who underwent complex segmentectomy of the lower lung lobe were selected. Among them, 42 patients of segmentectomy were guided by 3D reconstruction (3D group), while 55 were treated without this procedure (routine group). The demographics of patients and GGNs, such as age, sex, body mass index (BMI), preoperative pulmonary function, nodule size on LDCT, CTR, histology type, and comorbidity, were recorded. Written informed consent was obtained from all patients before operation.

### VATS segmentectomy procedure

Patients were placed in a lateral position after dual-lumen endotracheal intubation. The procedure of uniportal VATS segmentectomy was preferred with an incision of approximately 4–5 cm in the fourth or fifth intercostal space of the anterior axillary line. One or two additional ports may be needed if it is difficult to proceed with surgery in certain circumstances.

**Figure 2:**
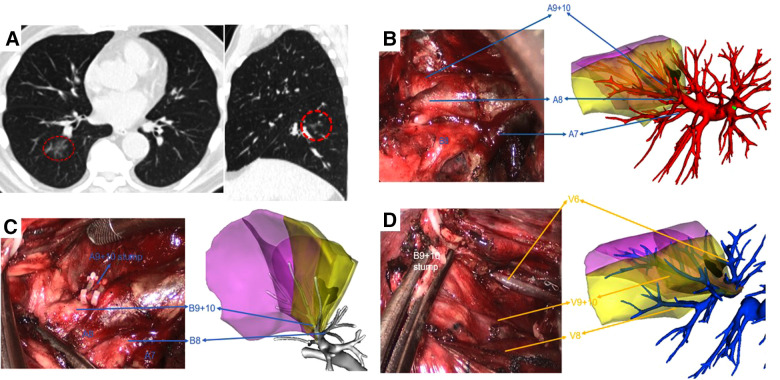
Right S9 + 10 VATS segmentectomy under real-time 3D reconstruction guidance. (**A**) preoperative CT scan showing a 10 mm GGN lesion in the right S9 + 10. (**B**) Branches of the pulmonary artery, (**C**) branches of the bronchus, and (**D**) branches of the pulmonary vein of the target segment were compared by using the real-time guidance of 3D reconstruction.

**Figure 3:**
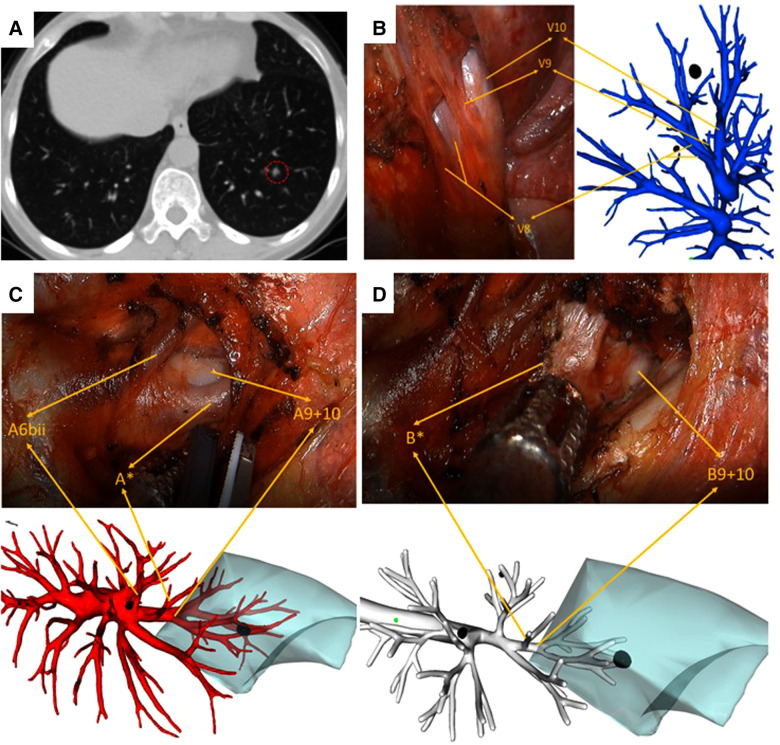
Left S9 VATS segmentectomy under real-time 3D reconstruction guidance (with the subsuperior segment S*). (**A**) preoperative CT scan showing a 6 mm GGN lesion in the left S9. (**B**) Branches of the pulmonary vein, (**C**) branches of the pulmonary artery, including A*, and (**D**) branches of the bronchus, including B*, compared by using the real-time guidance of 3D reconstruction.

All patients underwent the customary preoperative CT scan with a slice thickness of 1.0 mm. In the 3D reconstruction group, digital imaging and communications in medicine (DICOM) data for each patient were used for reconstruction with the software Vitaworks (Vitawork Medical technology Ltd, Shanghai, China). Surgeons isolated and identified the segmental artery, vein, and bronchus by comparing the 3D images with an iPad (or mobile phone) with an actual intraoperative situation repeatedly (Figures 2, 3). The bronchi were dissected by the stapler, while blood vessels were dissected by the stapler or hem-o-lock after confirmation. In the routine group, surgeons performed the surgery based on 2D CT images captured from the sagittal section, coronal section, and transverse section view.

A collateral ventilation method was used for delineating the intersegmental plane and subsequently resecting the target segment, as reported by a previous study (16). To reduce the postoperative air leak, staplers were used for transecting the intersegmental plane as early as possible. The resection margin of each nodule was examined by measuring the distance between the nodule and the resection margin. Generally, if the resection margin is less than 2 cm or the diameter of the nodule, an extra wedge resection would be executed for ensuring a safe surgical margin. Segmental, lobar, hilar, and lobe-specific mediastinal lymph node sampling or dissection (station 7–12) was performed for detecting primary lung cancer of the lower lung lobe. Lymph node sampling was performed for GGO with CTR ≤ 0.25. On the contrary, lymph node dissection was performed for GGO with CTR > 0.25. An absorbable polyglycolic acid felt (Neoveil, Guangzhou, China) was routinely used to cover the dissected bronchus and resection margin.

### Evaluation of perioperative outcomes

We counted the perioperative outcomes, including operation time, intraoperative blood loss, resection margins, the number of stapler reloads, postoperative drainage, postoperative hospital stay, and postoperative early complications. Postoperative early complication was defined as a complication occurring within 30 days from surgery, including postoperative air leakage on postoperative day (POD) 1–3 (chest tube observation), pneumonia/atelectasis (symptom and radiological examination), and hemoptysis (>10 ml). Postoperative complications were evaluated by using the Clavien–Dindo classification system (17).

### Statistical analyses

Statistical analyses were performed by using SPSS V.24.0 software (SPSS, Inc., Chicago, IL, USA). Continuous variables are presented as mean ± standard deviation, whereas categorical variables are presented as numbers and percentages. Chi-square test, Fisher’s exact test, or Student’s t-test was used to compare the differences between the variables. *P*-values <0.05 were considered statistically significant.

## Results

### Patient characteristics

The position and distribution of the complex segmentectomy of lower lung lobe are listed in Table 1. The characteristics of patients and lung nodules of the two groups are given in Table 2. In the 3D group, there were 10 males and 32 females. Thirty patients underwent uniportal VATS, while 12 patients underwent multiportal VATS during surgery. The mean age was 55.3 ± 13.2 years and BMI was 23.1 ± 3.6 kg/m^2^ of these patients. Preoperative ratio of forced expiratory volume in 1s to forced vital capacity (FEV1) was 83.7 ± 6.5% and ratio of diffusion lung capacity for carbon monoxide (DLCO) was 79.2 ± 8.2%. Nodule size on HRCT was 12.2 ± 4.4 mm and CTR of lung nodules less than 50% was detected in 33 patients. The routine group included 18 males and 37 females. Uniportal VATS was performed in 32 patients, while multiportal VATS was performed in 23 patients during surgery. The mean age was 59.4 ± 13.9 years and BMI was 23.1 ± 3.0 kg/m^2^ for the patients in this group. Preoperative FEV1 was 82.4 ± 7.7% and DLCO was 81.2 ± 10.0%. Nodule size on HRCT was 12.8 ± 4.5 mm and CTR of nodules less than 50% was detected in 41 patients. The most common histology type of pathology was adenocarcinoma in both groups. All of the baseline characteristics between the groups were comparable (all *P* > 0.05).

**Table 1:** The position and distribution of the complex segmentectomies of the lower lung.

Surgery types	3D group	Routine group
	(*n* = 42)	(*n* = 55)
Right lower lobe	23 (54.8%)	29 (52.7%)
S7	0	1
S8	11	9
S9	2	5
S10	2	5
S8 + 9	2	3
S9 + 10	4	4
Others	2	2
Left lower lobe	19 (45.2%)	26 (47.3%)
S8	6	6
S9	3	1
S10	4	10
S8 + 9	2	3
S9 + 10	2	5
Others	2	1

**Table 2:** The demographics of patients and lung nodules in the 3D group and routine group.

Values	3D group	Routine group	*P*-value
	(*n* = 42)	(*n* = 55)	
Age (years)	55.3 ± 13.2	59.4 ± 13.9	0.14
Sex			
Male	10	18	0.34
Female	32	37	
Pulmonary function			
FEV1 (%)	83.7 ± 6.5	82.4 ± 7.7	0.38
DLCO (%)	79.2 ± 8.2	81.2 ± 10.0	0.31
Nodule size on HRCT (mm)	12.2 ± 4.4	12.8 ± 4.5	0.46
CTR (%)			
0 ∼ 50	33	41	0.64
50 ∼ 100	9	14	
BMI (kg/m^2^)	23.1 ± 3.6	23.1 ± 3.0	0.93
Surgical approach			
Uniportal	30	32	0.18
Multiportal	12	23	
Histology type			0.7
Adenocarcinoma	38	51	
Squamous cell carcinoma	2	1	
Others	2	3	
Comorbidity			0.78
Hypertension	10	9	
Diabetes	5	4	
Coronary artery disease	5	7	

CTR, consolidation-to-tumor ratio; HRCT, high-resolution computer tomography.

### Perioperative outcomes

There was no 30-day postoperative mortality and conversion in the two groups. Perioperative outcomes are outlined in Table 3. The operation time of the 3D group (111.4 ± 20.8 min) was significantly shorter than that of the routine group (127.1 ± 32.3 min) (*P* = 0.007). The number of stapler reloads during surgery in the 3D group was less than that in the routine group (9.0 ± 2.2 vs. 10.4 ± 2.6, *P* = 0.009). The rate of air leakage on postoperative day 1–3 was lower in the 3D group (11.9% vs. 30.9%, *P* = 0.03). In addition, the resection margin of all the patients in the 3D group was safe and adequate, while four patients in the routine group had inadequate resection margins, although there was no statistically significant difference (*P* = 0.13).

**Table 3:** Perioperative outcomes of the 3D group and routine group.

Values	3D group	Routine group	*P*-value
	(*n* = 42)	(*n* = 55)	
Operation time (min)	111.4 ± 20.8	127.1 ± 32.3	0.007
Intraoperative blood loss (ml)	47.9 ± 29.1	51.1 ± 36.3	0.638
Inadequate resection margins	0 (0%)	4 (7.3%)	0.131
Number of stapler reloads	9.0 ± 2.2	10.4 ± 2.6	0.009
Postoperative drainage (ml)	488.7 ± 188.4	525.0 ± 229.3	0.407
Postoperative hospital stay (days)	4.6 ± 1.7	4.4 ± 1.7	0.521
Postoperative complications			
Air leakage on POD 1–3	5 (11.9%)	17 (30.9%)	0.027
Pneumonia/atelectasis	4 (9.5%)	4 (7.3%)	0.979
Hemoptysis (>10 ml)	2 (4.8%)	4 (7.3%)	0.934

Inadequate surgical margin: resection margin <2 cm or the diameter of the nodule.

POD, postoperative day.

Intraoperative blood loss in the 3D group was 47.9 ± 29.1 ml, compared with 51.1 ± 36.3 ml in the routine group (*P* = 0.64). In addition, postoperative drainage (488.7 ± 188.4 vs. 525.0 ± 229.3 ml, *P* = 0.41), postoperative hospital stay (4.6 ± 1.7 vs. 4.4 ± 1.7 days, *P* = 0.52), pneumonia/atelectasis (4 vs. 4, *P* = 0.98), and hemoptysis (2 vs. 4, *P* = 0.93) were comparable between the two groups.

## Discussion

A recent meta-analysis has shown that preoperative 3D lung simulation could achieve better intraoperative and postoperative outcomes in terms of blood loss, operative time, conversion rate, postoperative hospital stay, and complications (18). However, relevant studies were seldom exclusive for exploring the role of 3D reconstruction in thoracoscopic complex segmentectomy of the lower lung lobe.

The segment anatomic variation of the lower lung lobe is more common than that of the upper lung. The subsuperior segment (S*), which is located between the superior and the basal segments, has been rarely mentioned by researchers during the past few years. With 3D reconstruction, it is more convenient to identify each pulmonary lobe, especially for this atypical pulmonary segment. Recent literature has reported the prevalence of subsuperior segments ranging from 24% to 32.04%, which indicates that subsuperior segments were not very rare in clinical work (19, 20). Usually, the subsuperior segment is difficult to identify by using the routine CT images due to its tiny bronchus (B*), artery (A*), and vein (V*). It is here 3D reconstruction plays an immensely advantageous role by displaying precisely all the structures of vessels and bronchi. The patient, as depicted in Figure 3, in our study had S* variation, and we successfully completed the surgery by depending on 3D reconstruction images. S* segmentectomy may be appropriate if a pulmonary nodule is located exactly in the center of S*. However, a combined segmentectomy such as S6 + S* or S10 + S* is unnecessary if the nodule is located on the border of S* and other basal segments (21). Therefore, preoperative identification of S* by 3D reconstruction is extremely important for realizing a precise segmentectomy.

Lower pulmonary arteries and veins may have minor anatomic variations, or even no variations (15). A 3D reconstruction before the performance of segmentectomy could effectively distinguish the anatomic relationship and variation of blood vessels and bronchi. It could build a spatial model that is similar to an operative setting, which is difficult to obtain from 2D CT images. In addition, reconstructed images could be rotated freely from any angle, which is beneficial for preoperative simulated training. For performing complex segmentectomy, it may have greater benefits. Preoperative 3D-CT images make it easier for thoracic surgeons to recognize precisely the vascular and bronchi branches of the target segment during surgery and, therefore, shorten the operation time. Our study found that the mean operation time in the 3D group was significantly shorter than that in the routine group (111.4 ± 20.8 min vs. 127.1 ± 32.3 min, *P* < 0.01), which was consistent with the conclusion provided by Xue et al. in their study (10).

Postoperative air leak prolongs hospital stay and increases hospitalization expenditures during the performance of segmentectomy (22). A 3D reconstruction before performing segmentectomy may prevent the incidence of air leak. Wu et al. found that air leakage on postoperative day 1 in the 3D group was higher than that in the routine group (56.4% vs. 16.4%, *P* = 0.020) (7). However, we drew opposite conclusions from our study (11.9% in the 3D group vs. 30.9% in the routine group, *P* = 0.03). This may be partly explained by the fact that 3D reconstruction could precisely identify the anatomic structures and shorten operation time, thus reducing the intraoperative redundant rotation and chance of injury of lung tissues.

Sufficient resection margin during segmentectomy is indispensable for decreasing the local recurrence rates and improving the survival time (23). In our study, we examined the resection margin of each nodule by measuring the distance between the nodules and the resection margins. Eventually, we found that the surgical margins of four patients in the routine group were inadequate, and therefore, we performed additional wedge resection in these patients. On the other hand, all patients in the 3D group had adequate resection margins. We speculated that 3D reconstruction could provide the precise location of each lung nodule and guarantee the exact dissection of blood vessels. If the feed vessels of the targeted segment could not be identified correctly, the inflation–deflation technique would reveal an inaccurate intersegmental plane, resulting in inadequate resection margins.

More stapler reloads during surgery increase the hospital cost. In our study, we observed that the number of stapler reloads in the 3D group was less than that in the routine group [9.0 ± 2.2 vs. 10.4 ± 2.6 (*P* < 0.01)]. The reasons may be as follows. First, 3D reconstruction helps provide a better dissection plan of targeted segments. Second, four patients with inadequate resection margins in the routine group inevitably increased the number of stapler reloads after additional wedge resection.

There are some limitations in our study. First, it is a retrospective study with a small sample size and single center. A multicenter, large-sample randomized controlled trial is expected to validate our conclusion. Second, long-term follow-up and postoperative pulmonary function results are lacking in this study.

In summary, 3D reconstruction during the performance of segmentectomy has significant advantages in terms of locating nodules and identifying the variations of vessels and bronchi. For complex segmentectomy of the lower lung lobe, 3D reconstruction may shorten the operation time, decrease the number of stapler reloads, prevent postoperative air leakage, and guarantee a safe surgical margin. Therefore, 3D reconstruction is recommended for complex segmentectomy of the lower lung lobe.

## Data Availability

The data that support the findings of this study are available on request from the corresponding author.
